# Idiopathic dendriform pulmonary ossification as the phenotype of interstitial lung abnormalities: CT–pathologic correlation and prevalence

**DOI:** 10.1007/s11604-024-01590-8

**Published:** 2024-05-14

**Authors:** Midori Ueno, Ryoko Egashira, Mikiko Hashisako, Kiminori Fujimoto, Taiki Fukuda, Yoshiko Hayashida, Hiromitsu Sumikawa, Junya Tominaga, Tomonori Tanaka, Yasuhiro Terasaki, Junya Fukuoka, Yasuhiko Nishioka, Takatoshi Aoki, Toshifumi Gabata, Hiroto Hatabu, Takeshi Johkoh

**Affiliations:** 1 Department of Radiology, University of Occupational and Environmental Health School of Medicine, 1-1 Iseigaoka, Kitakyushu City, Fukuoka 807-8555 Japan; 2 Department of Radiology, Faculty of Medicine, Saga University, 5-1-1, Nabesima, Saga City, Saga 849-8501 Japan; 3 Department of Anatomic Pathology, Pathological Sciences, Graduate School of Medicine Sciences, Kyushu University, 3-1-1, Maedashi, Fukuoka City, Fukuoka 812-8582 Japan; 4 Department of Radiology, Kurume University School of Medicine, 67, Asahimachi, Kurume City, Fukuoka 830-0011 Japan; 5 Department of Radiology, The Jikei University School of Medicine, 3-25-8, Nishishinbashi, Minato-Ku, Tokyo, 105-8461 Japan; 6 Department of Radiology, National Hospital Organization Kinki-Chuo Chest Medical Center, 1180, Nagasonecyo, Kita-Ku, Sakai City, Osaka 591-8555 Japan; 7 Department of Diagnostic Radiology, Tohoku University School of Medicine, 1-1, Seiryoucyo, Aoba-Ku, Sendai City, Miyagi 980-8574 Japan; 8 Department of Diagnostic Pathology, Kobe University Hospital, 7-5-2, Kusumachi, Cyuoh-Ku, Kobe City, Hyogo 65017 Japan; 9 Department of Analytic Human Pathology, Nippon Medical School Hospital, 1-1-5, Sendaki, Bunkyo-Ku, Tokyo, 113-8603 Japan; 10 Department of Pathology Informatics, Nagasaki University Graduate School of Biomedical Sciences, 1-7-1, Sakamoto, Nagasaki City, Nagasaki 852-8501 Japan; 11 Department of Respiratory Medicine and Rheumatology, Graduate School of Biomedical Sciences, Tokushima University, 2-50-1, Kuramotocyo, Tokushima City, Tokusima 770-0042 Japan; 12 Department of Radiology, Kanazawa University Graduate School of Medical Science, 13-1, Takaramachi, Kanazawa City, Isihikawa 920-8641 Japan; 13 Department of Radiology, Brigham and Women’s Hospital, Harvard Medical School, 75, Francis Street, Boston, 02115 USA; 14 Department of Radiology, Kansai Rosai Hospital, 3-1-69, Inabaso, Amagasaki City, Hyogo 660-0064 Japan

**Keywords:** Dendriform pulmonary ossification, Radiologic–pathologic correlation, Interstitial lung abnormalities, Cicatricial organizing pneumonia, Thin-section CT

## Abstract

**Background and purpose:**

Idiopathic dendriform pulmonary ossification (DPO) is mostly asymptomatic, and detected incidentally in lung CT. There have been no reports on the precise CT–pathologic correlation and the prevalence of idiopathic DPO. This study aimed to clarify the histological background and prevalence of idiopathic DPO.

**Materials and methods:**

Sixteen patients with histologically confirmed idiopathic DPO (12 men and 4 women; mean age, 38.8 years; range 22–56 years) were identified in a nationwide epidemiological survey. Local HRCT findings of pre-biopsy examinations, such as branching, round, linear structures with or without high attenuation were compared side by side with histological findings. The attenuation of branching, round, and linear structures was classified into three-point levels on bone window images (width, 2500 HU; level, 500 HU). Furthermore, we collected continuous pulmonary CT images of 8111 cases for checking up metastasis from extrathoracic malignancy at a single institution, and evaluated the prevalence of interstitial lung abnormalities (ILAs) and DPO.

**Results:**

In all 16 cases, branching (*n* = 15, 93%), round (*n* = 5, 31%), or linear (*n* = 5, 31%) structures were identified, histologically corresponding to dendriform ossification and cicatricial organizing pneumonia (OP)/fibrosis. Histologically, ossification was confirmed in all the 16 patients. However, in two cases, a highly attenuated structure could not be detected on the pre-biopsy CT of the same area. Regarding the prevalence of idiopathic DPO, 283 (3.5%) of 8111 patients had ILAs, of which a total of 26 (0.3% of all cases, 9.2% of ILAs cases) had DPO.

**Conclusion:**

Idiopathic DPO showed linear or branching structures with or without high attenuation on CT, corresponded to ossification, cicatricial OP/fibrosis. DPO was seen in 9.2% of ILAs cases. Idiopathic DPO is one of pathologic phenotypes of ILAs.

**Supplementary Information:**

The online version contains supplementary material available at 10.1007/s11604-024-01590-8.

## Introduction

Interstitial lung abnormalities (ILAs) are defined as incidental CT findings of non-dependent abnormalities, including ground-glass opacities or reticular abnormalities, lung distortion, traction bronchiectasis, honeycombing, and non-emphysematous cysts, those affecting more than 5% of any lung zone [1]. According to the previous report, majority of ILAs were considered to be pathological early usual interstitial pneumonia [2]. However, whole pathological backgrounds of ILAs remain unclear.

Diffuse pulmonary ossification is a rare and mostly asymptomatic condition characterized by metaplastic ossification leading to small bone fragments in the lung tissue. The clinical course of this disease is indolent or slowly progressive [3], and it is sometimes detected incidentally in lung CT in our experience. Thus, diffuse pulmonary ossification is one of the candidates of pathological phenotypes in ILAs.

Clinically, diffuse pulmonary ossification can be classified into idiopathic or secondary ones. It is commonly seen in chronic respiratory diseases, such as idiopathic pulmonary fibrosis (IPF) and chronic obstructive pulmonary disease. Recently, DPO has been described as a part of histological findings of cicatricial organizing pneumonia (OP), which is a variant of OP [4, 5]. The morphology of ossification has two patterns: dendriform and nodular pulmonary ossification, and idiopathic diffuse pulmonary ossification is thought to be dendriform [6]. Idiopathic dendriform pulmonary ossification (DPO) is rare and only a few cases have been reported to date. Moreover, there are few comprehensive reports on imaging findings of idiopathic DPO [6–11]. In idiopathic DPO, despite the absence of background respiratory diseases, such as idiopathic pulmonary fibrosis, HRCT shows linear, reticular, and nodular shadows mixed with microcalcification and ossification. However, it is not clear which histological findings correspond to CT findings in idiopathic DPO. Furthermore, the prevalence of DPO and especially that in ILAs remains unclear.

The objectives of the present study were twofold: the first one is to clarify the histological background of HRCT findings in idiopathic DPO by side-by-side CT–pathologic correlation, and the second one is to find the prevalence of idiopathic DPO and especially that in ILAs.

## Materials and methods

This study was conducted as a part of the Radiology and Pathology Collaboration Project, Pathology and Cryobiology Section, Research Group on Diffuse Pulmonary Diseases, Policy Research Project for Intractable Diseases, and Grant-in-Aid for Health, Labor and Welfare Science Research in Japan in FY2020. This study was approved by the Committee on Human Ethics of the Primary Investigation Institute (Approval Nos. 3084 and 3251). This retrospective study used existing data and waived the requirement for informed consent.

### Precise CT–pathologic correlation

#### Data source and case selection

**Fig. 1:**
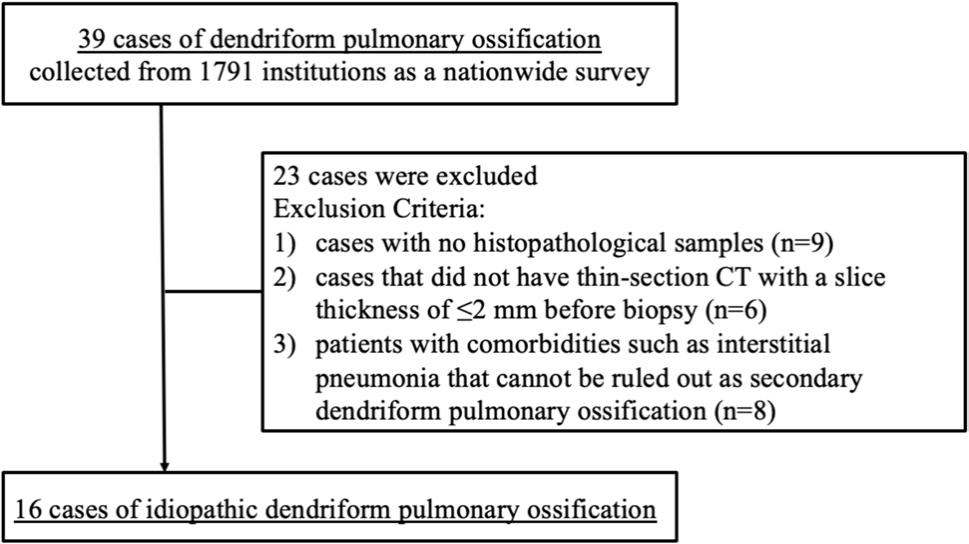
Participant flow diagram. 39 cases of pulmonary dendriform ossification were retrospectively collected from 1791 institutions as a nationwide survey. From these 39 cases, we excluded those that met either following criteria: (1) cases with no histopathological samples, (2) cases that did not have thin-section CT with a slice thickness of ≤ 2 mm before biopsy, and (3) patients with comorbidities such as interstitial pneumonia that cannot be ruled out as secondary. Finally, we evaluated 16 cases of idiopathic DPO

DPO cases were collected as a nationwide survey of the Diffuse Alveolar Microlithiasis and Pulmonary Ossification Section in Policy Research Project for Intractable Diseases performed from 2017 to 2019 [6]. In the database, 39 cases were retrospectively collected from 1791 institutions. From these 39 cases, we excluded those that met either following criteria: (1) cases with no histopathological samples, (2) cases that did not have thin-section CT with a slice thickness of ≤ 2 mm before biopsy, and (3) patients with comorbidities such as interstitial pneumonia that cannot be ruled out as secondary. Finally, we evaluated 16 cases of idiopathic DPO (Fig. 1). Although the patients overlapped with the prior report [6], the target of the present study is different, as the previous report evaluated the clinical characteristics of idiopathic DPO, and the current study focused on the side-by-side comparison of local CT findings at the surgical lung biopsy resection site, and histological images and the prevalence of DPO in another cohort.

#### Radiologic examination

All CT images were obtained in the supine position with deep inspiration. Because of the multi-institutional retrospective nature of this study, scanning protocols were not standardized. The thinnest slice thicknesses of the available CT images were less than or equal to 1 mm in 14 cases, and 1.25 mm and 2 mm in the remaining two cases, respectively.

#### Image analysis

Two thoracic radiologists with 14 and 8 years of experiences, respectively, evaluated the CT findings at the surgical lung biopsy resection site. The display conditions for CT images were WW 2500 HU and WL 500 HU for bone window settings and WW 1400 HU and WL -500 HU for lung window settings.

**Fig. 2:**
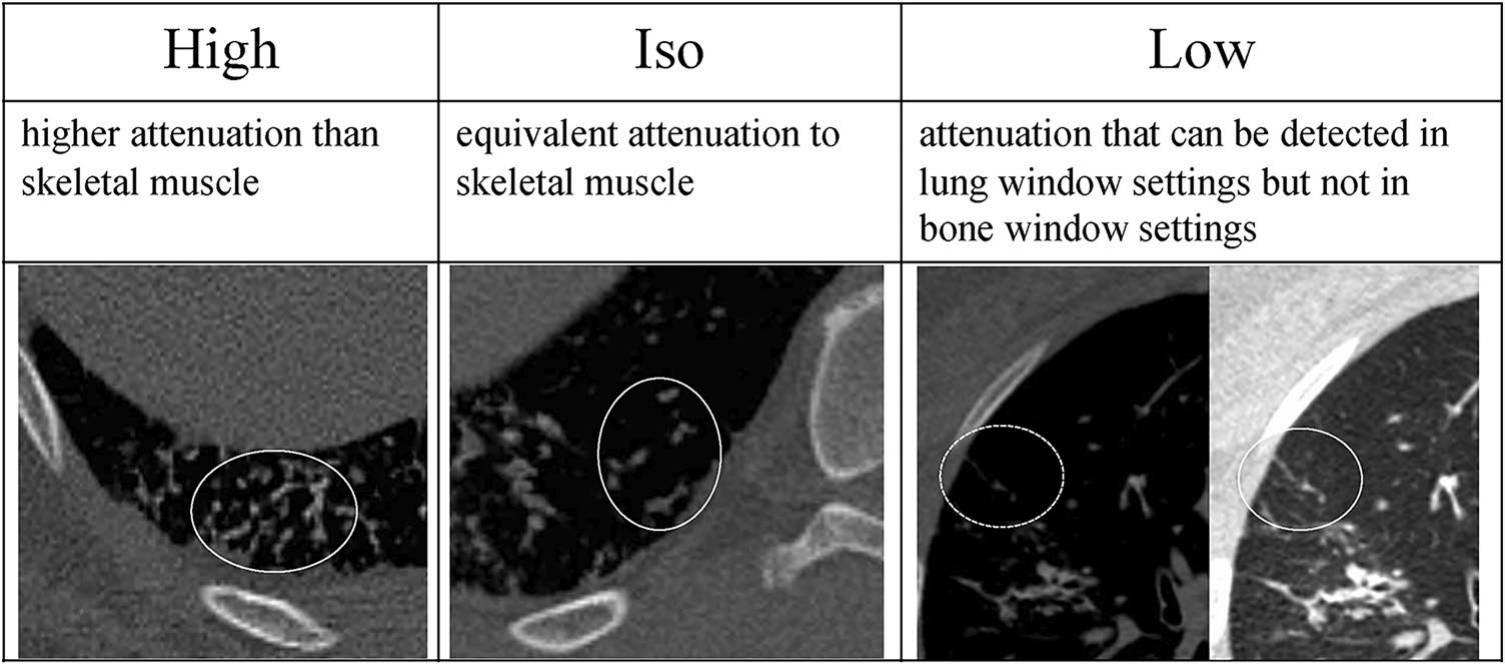
CT attenuation level at bone window settings (WW 2500HU, WL 500HU). After evaluating the presence of branching, linear, and round structures in lung window settings, the attenuation values of these structures were classified into three levels in bone window settings (WW 2500HU, WL 500HU): high, higher attenuation than skeletal muscle; iso, equivalent attenuation to skeletal muscle; and low, attenuation that can be detected in lung window settings but not in bone window settings (shown in the circle of each image). Figure 5 shows a representative case of “high”, and Fig. 6 shows the representative case of “iso”

First, the presence or absence of branching, linear, and round structures was evaluated in the lung window settings. Second, the attenuation was classified into three-point levels in the bone window settings: “high,” higher attenuation than skeletal muscle; “iso,” attenuation equivalent to skeletal muscle; and “low,” attenuation that can be pointed out in the lung window settings but cannot be pointed out in the bone window settings (Fig. 2). When two or more attenuation levels coexisted in one continuous structure, it was also classified as “mix”. In addition, when a highly attenuated structure could not be detected in the bone window settings, additional evaluation was performed in the osteoporosis window settings (WW 818 HU, WL 273 HU) reported to be highly sensitive to detect pulmonary ossification on CT [12]. The presence or absence of other findings such as band-like opacity, areas with ground-glass attenuation (GGA), lobules with decreased attenuation, reticulation, cysts, and pleural indentation, and distribution (craniocaudal, transaxial, anteroposterior) were also analyzed in lung window settings. Detailed definitions of each term are provided in the Electronic Supplemental Materials [7–11, 13–15]. Each radiologist independently evaluated CT images, and discrepancy was finalized by mutual agreement.

Interobserver agreement between the two radiologists was assessed by calculating both the *κ* value and a simple agreement concerning the presence or absence of each CT findings. We used the following scale for the evaluation of agreement by *κ* value: 0–0.20, poor agreement; 0.21–0.40, fair agreement; 0.41–0.61, moderate agreement; 0.61–0.80, good agreement; > 0.81, excellent agreement.

#### Pathologic examination and precise CT–pathologic correlation

Biopsy specimens were available for all the 16 patients. The presence of ossification, cicatricial OP [4], fibrosis, hemorrhage, dilation of the alveolar duct, emphysema, and pleural thickening was evaluated. If ossification was present, the long diameter and location of the ossification on the specimen, presence of bone marrow, and relationship to cicatricial OP or fibrosis were evaluated. Cicatricial OP was defined as a variant of organizing pneumonia in which the airspaces of the lung were filled with and consolidated by dense collagenized scar tissue associated with preservation of underlying lung architecture [4].

CT–pathologic correlation was performed using surgical biopsy specimens by one thoracic pathologist with eight-years of experience and four thoracic radiologists with 8, 14, 21, and 33 years of experience, respectively. The resection site on the pre-biopsy CT was identified by referring to the information from each institution and the resection scar on the post-biopsy CT. CT findings at the biopsy resection site and pathological findings were evaluated in each case to determine which pathological findings were reflected in the CT findings. Decisions were made by consensus.

### Abstraction of prevalence of a cohort of screening metastasis from extrathoracic malignancies

During the period from April 2020 to March 2023, postoperative patients with extrathoracic malignancy who underwent screening CT for lung metastases more than 6 months after surgery at single institution were included in the evaluation. In cases in which multiple CTs were performed within the period, the first CT scan within the period was evaluated. Patients with respiratory symptoms or those already diagnosed with pulmonary disease were excluded.

One thoracic radiologist with 33-year experience assessed the CT images for the presence of ILAs and if present, the pattern of ILAs (i.e., non-subpleural, subpleural non-fibrotic, or subpleural fibrotic), and whether it was DPO or not. ILAs were defined as the incidentally detected CT findings in patients without previous clinical suspicion of interstitial lung disease (ILD); they are described as non-dependent abnormalities affecting more than 5% of any lung zone [1]. From the present and previous studies [6–10], DPO on CT was defined as lower lobe predominant linear, branching, or dot-like structures with calcification at least in one lesion. We did not include calcification that could clearly be determined to be another pathology, such as calcified granulomas or calcification of the cartilage wall, in DPO.

CT images were obtained using multislice CT scanners at single institution (Aquilion One, Canon medical, Japan). Whole lung volume data of 0.5 mm collimation was acquired by iterative reconstruction. The data obtained were reconstructed with a 1 mm thickness (using a high-spatial-frequency algorithm) or 5 mm thickness (using a standard algorithm).

## Results

### Precise CT–pathologic correlation

#### Patients’ background

Sixteen patients (12 men, 4 women; mean age, 38.8 years; range, 22–56 years) were included in this study. The patients’ age, sex, and clinical background are shown in Table 1.

**Table 1:** Clinical features of 16 cases of idiopathic dendriform pulmonary ossification

Case	Age	Sex	Medical history
1	27	M	Sleep apnea syndrome
2	51	W	N/A
3	47	M	N/A
4	46	M	Coronary spastic angina、hypertension
5	29	M	Gilbert syndrome, sinus tachycardia
6	43	M	Hypertension, reflux esophagitis
7	43	M	Atopic dermatitis, alcoholism
8	56	W	N/A
9	48	W	Diabetes mellitus (type 1), Dactylosymphysis, schizophrenia
10	26	M	N/A
11	32	M	N/A
12	33	M	Dyslipidemia, hyperuricemia
13	35	M	N/A
14	22	M	Allergic rhinitis
15	43	W	N/A
16	39	M	Hypertension, primary aldosteronism

*M* man, *W* woman, *N/A* not applicable

#### CT findings

The CT findings of the 16 patients with idiopathic DPO are shown in Table 2. Interobserver agreements for the determination of the CT features and attenuation level are listed in the Electronic Supplemental Table 1, and are mostly good (good to excellent agreement), except for areas with GGA.

**Table 2:** CT findings of 16 cases of idiopathic dendriform pulmonary ossification

CT findings	Number of cases	%
Branching	15	93
High	14	87
Iso	15	93
Low	5	31
Mixed	15	93
Round	5	31
High	0	0
Iso	5	31
Low	1	6
Mixed	0	0
Linear	5	31
High	0	0
Iso	5	31
Low	2	12
Mixed	1	6
Band-like opacity	3	18
Pleural indentation	8	50
Lobules with degreased attenuation	7	43
Ground-glass attenuation (GGA)	1	6
Reticulation	0	0
Cysts	0	0
Craniocaudal distribution		
Upper predominance	0	0
Lower predominance	11	69
Random	5	31
Transaxial distribution		
Central predominance	0	0
Peripheral predominance	6	37
Random	10	63
Anteroposterior distribution		
Anterior predominance	0	0
Posterior predominance	11	69
Random	5	31

In all 16 cases, branching (*n* = 15, 93%), round (*n* = 5, 31%), or linear (*n* = 5, 31%) structures were identified at the biopsy sites of the pre-biopsy CT images in the lung window setting, and these findings were observed in the centrilobular and perilobular areas. These branching, round, and linear structures were classified as high, iso, or low according to the attenuation value in the bone window settings. The frequency of each form for each attenuation level is presented in Table 2. Fourteen of the branching form structures were high, 15 were iso, and five were low, with iso and high attenuation levels being the most common. In round structures, five cases were iso, one case was low, and iso was the most common attenuation level. Linear structures were observed in five cases and were low in two cases, with iso-attenuation levels being the most common. When classified by attenuation level, 14 of the 16 cases were high, 16 were iso, and 8 were low, regardless of the structure type. In two of the 16 cases, no high attenuation structure was identified on CT corresponding to the biopsy site in bone window settings. In these two cases, additional evaluation was performed in the osteoporotic window setting [10], and no high attenuation structure was identified. The slice thickness in those two cases was 1.25 mm in one case and 1 mm in the other.

Other findings included pleural indentation in eight cases, lobules with decreased attenuation in seven cases, band-like opacity in three cases, and areas with GGA in one case in the lung window settings. For the craniocaudal, transaxial, and anteroposterior distribution, 11 of 16 cases were lower predominance, 10 cases were peripheral predominance, and 11 cases were posterior predominance.

#### Histological findings

Histological findings are presented in Table 3. Branching and nodular ossification were observed in all the 16 patients. The long diameter of the ossification in the specimens ranged from 0.1 mm to 6 mm. The bone marrow was identified in 15 patients (93%). The location of ossification was difficult to recognize in advanced areas with tissue destruction and contraction, and evaluations were performed in areas of the lungs with limited structural destruction. In all 10 cases in which the location of ossification was recognized, ossification was located in air spaces, such as alveolar ducts, and bone attachments were found in the interstitium/alveolar septum.

**Table 3:** Histological findings of 16 cases of idiopathic dendriform pulmonary ossification

Histological findings	Number of cases	%
Multiple foci of ossification in a nodular and branching pattern	16	100
Bone marrow	15	93
Cicatricial organizing pneumonia	15	93
Pleural thickening	14	87
Dilatation of alveolar duct	12	75
Emphysema	8	50
Hemorrhage	6	37
Fibrosis	5	31

**Fig. 3:**
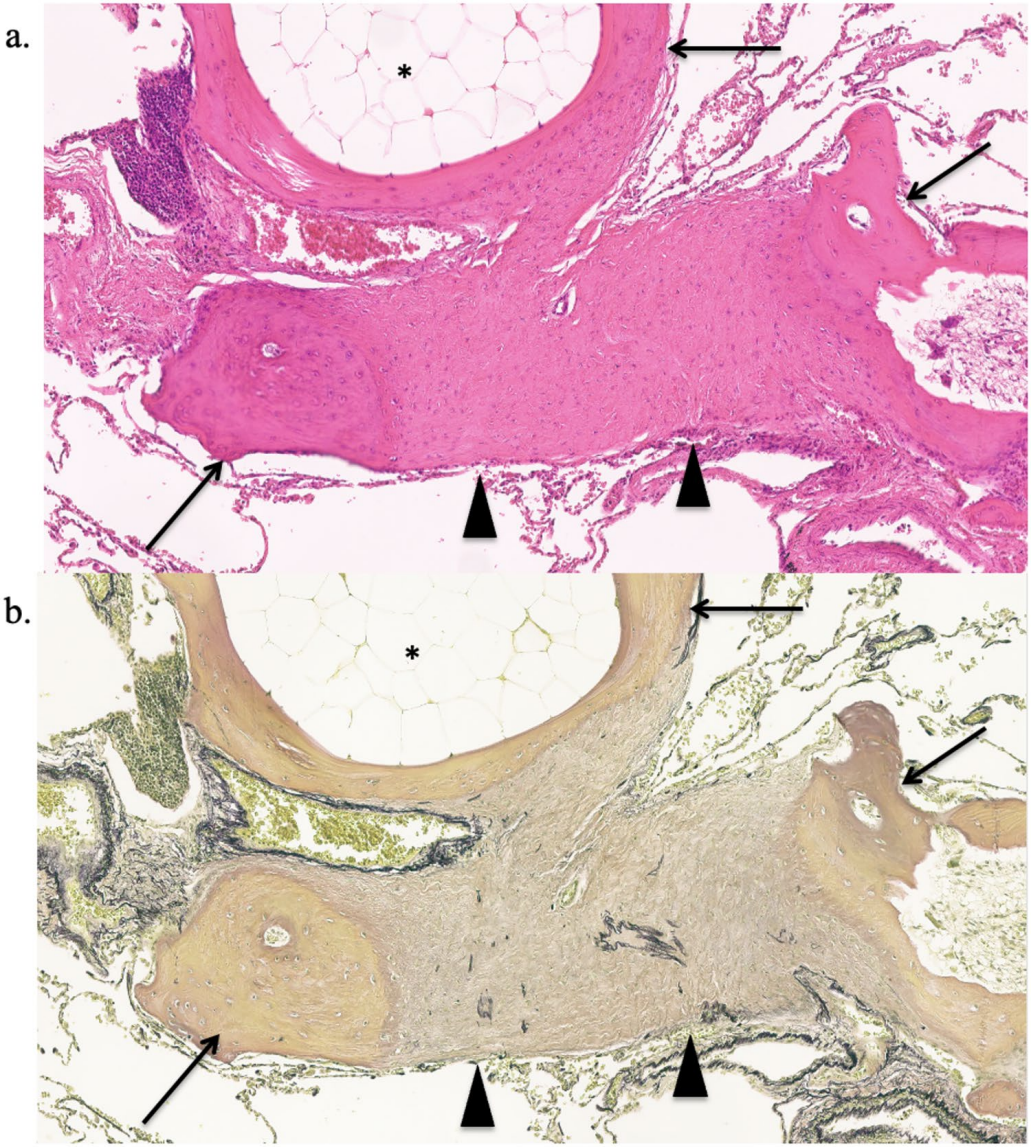
A 47-year-old man with idiopathic DPO. Photomicrograph showing ossification (arrows) located at the margin of the cicatricial OP (arrowheads); the cicatricial OP is broadly adjacent to the ossification. Part of the ossification contains fat marrow (asterisk) [**a** magnification ×100, hematoxylin–eosin (HE) stain; **b** ×100; EVG stain]

Cicatricial OP was observed in 15 of 16 cases (93%), and cicatricial OP was distributed in airspaces such as alveolar ducts and alveolar space in all 15 cases. Fourteen cases (87%) showed ossification at the margins of the cicatricial OP, and ossification and cicatricial OP were broadly adjacent to each other (Fig. 3). In addition, pleural thickening was observed in 14 cases (87%), dilatation of the alveolar ducts in 12 (75%), emphysema in eight cases (50%), and hemorrhage in six cases (37%).

#### CT–pathologic correlation

**Fig. 4:**
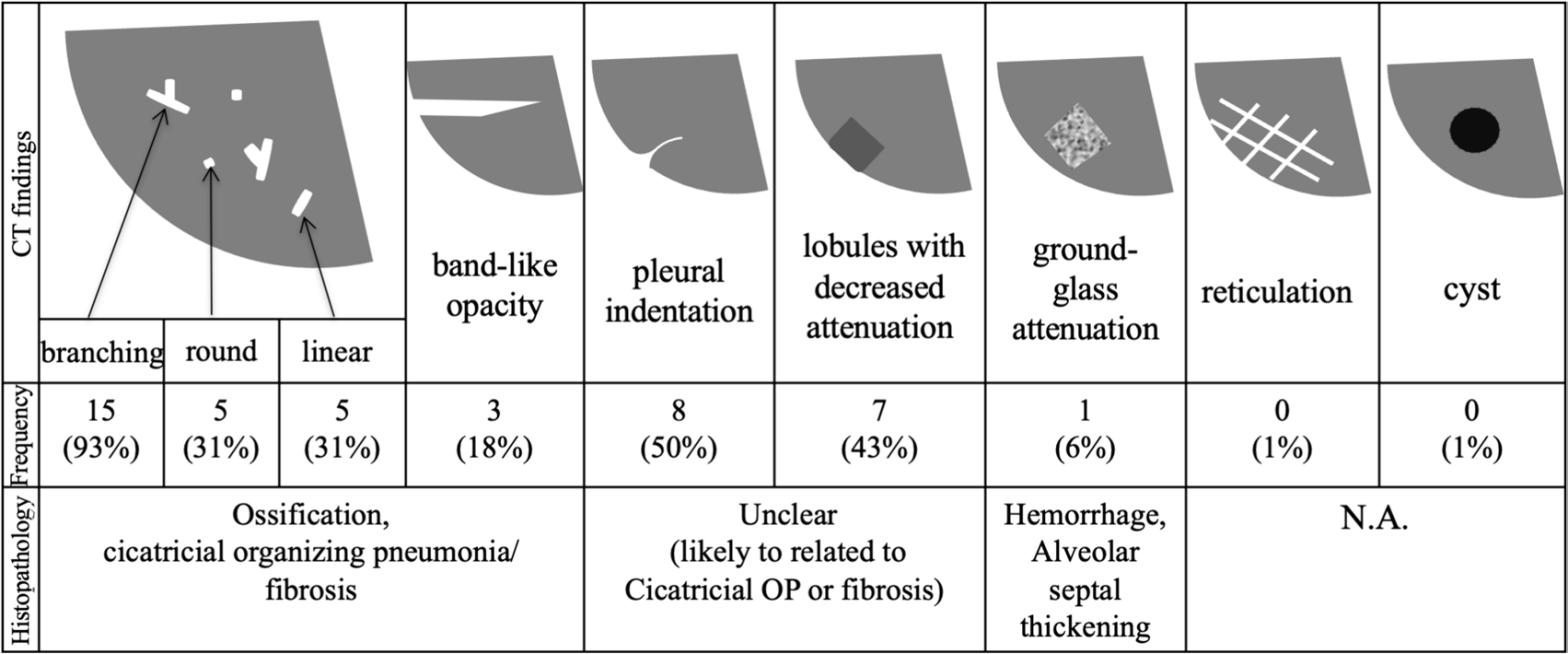
Radiologic findings of biopsy sites of pre-biopsy CT images and CT–pathologic correlation. The branching, linear, and round structures seen in the CT lung window settings reflected ossification, cicatricial OP, and more mature fibrosis than OP observed in the pathology. Band-like opacity reflected a slightly larger cicatricial OP and partial ossification, and ground-glass attenuation reflected hemorrhage and alveolar septal thickening. The pathological findings corresponding to pleural indentation and hypoattenuated lobules could not be clearly identified. Reticulation and cyst are histopathologically not applicable (N.A.). Figure 5 shows a representative case of “branching”, Fig. 6 shows the representative case of “round”, and Fig. 7 shows the representative case of “band-like opacity”

**Fig. 5:**
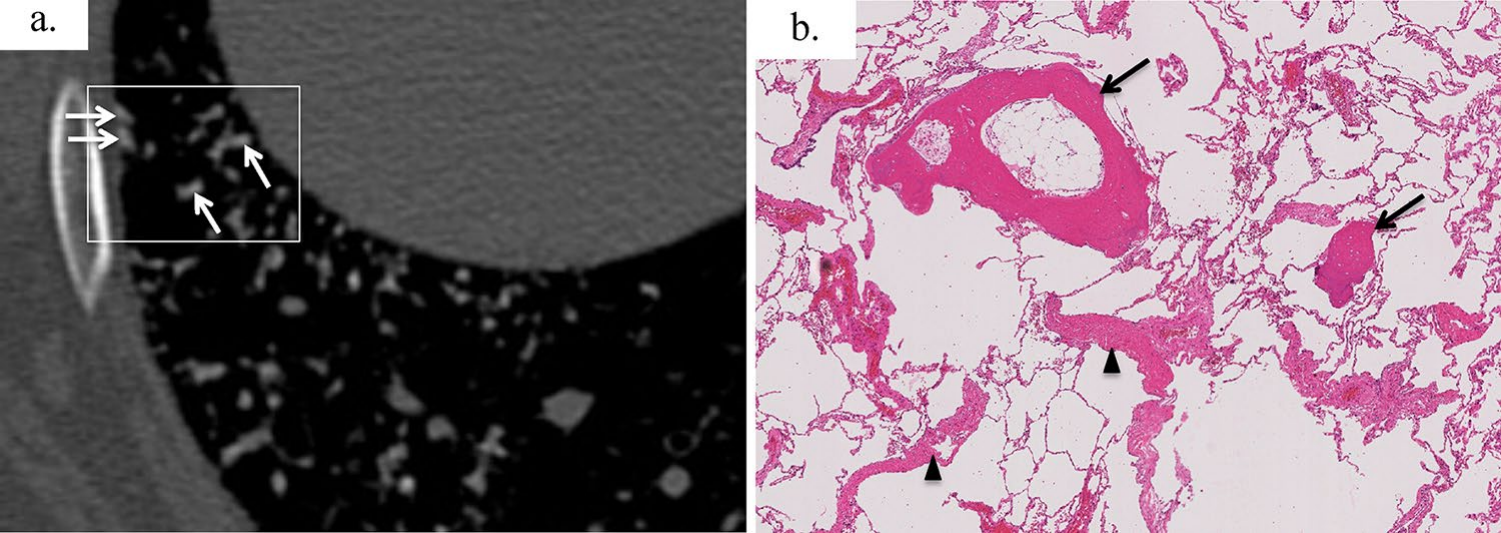
Precise CT–pathologic correlation of a 43-year-old woman with idiopathic DPO; the side-by-side comparison of local CT findings at the surgical lung biopsy resection site indicated by a white square (Fig. 5a) and histological images. **a** Unenhanced axial chest CT in lung window settings showing highly attenuated branching structures (arrows). **b** Surgical lung biopsy specimen showing multiple nodular and branching ossifications (arrows) mainly in the alveolar duct to the alveoli. Ossification involves fatty bone marrow. Emphysema and scattered foci of cicatricial OP (arrowheads) were also noted (HE eosin staining)

**Fig. 6:**
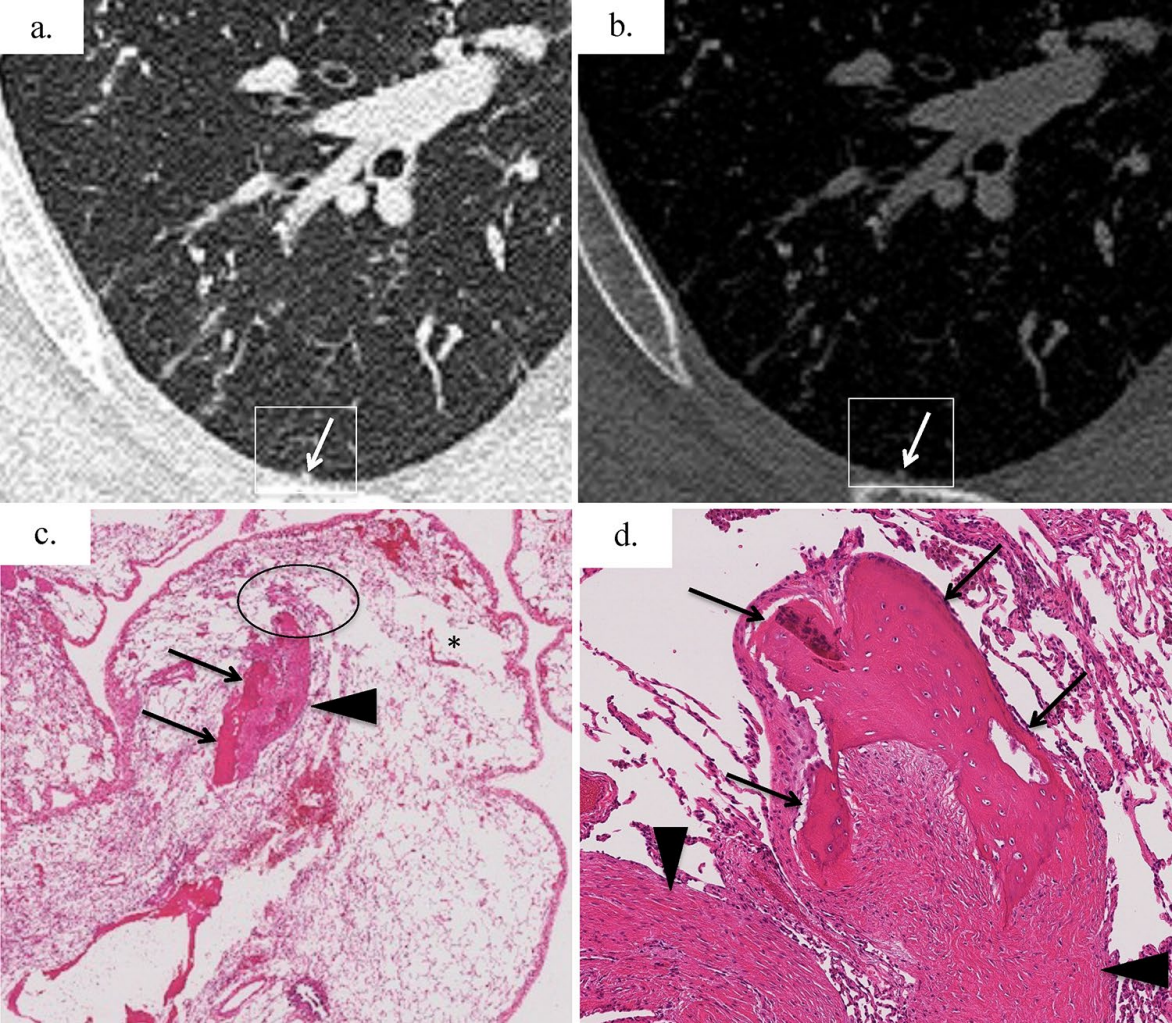
Precise CT–pathologic correlation of a 39-year-old man with idiopathic DPO; the side-by-side comparison of local CT findings at the surgical lung biopsy resection site indicated by a white square (**a**, **b**) and histological images. **a** Unenhanced axial chest CT at lung window settings showing a tiny round structure (arrow) in the subpleural area. **b** The tiny round structure is iso-attenuated in bone window settings. In this case, a highly attenuated structure was not observed at the exact site of the pre-biopsy CT images. **c** Histologically, the section shows ossification (arrows) at the margin of the cicatricial OP (arrowhead). Localized mild fibrotic thickening of the alveolar septa (circle) and emphysema (asterisk) are also present (HE stain). **d** Cicatricial OP (arrowheads) and ossification (arrows) are broadly adjacent to each other (× 100, HE stain)

A summary of the CT pathological correlation is shown in Fig. 4. Branching, round, and linear structures histologically corresponded to ossification, cicatricial OP, or fibrosis. These structures showed various attenuation levels of high, iso, and low in the bone window settings (Fig. 5). Bone formation was confirmed in all 16 histopathological specimens. However, in two cases, no high-attenuation area was identified at the biopsy site on CT (Fig. 6).

**Fig. 7:**
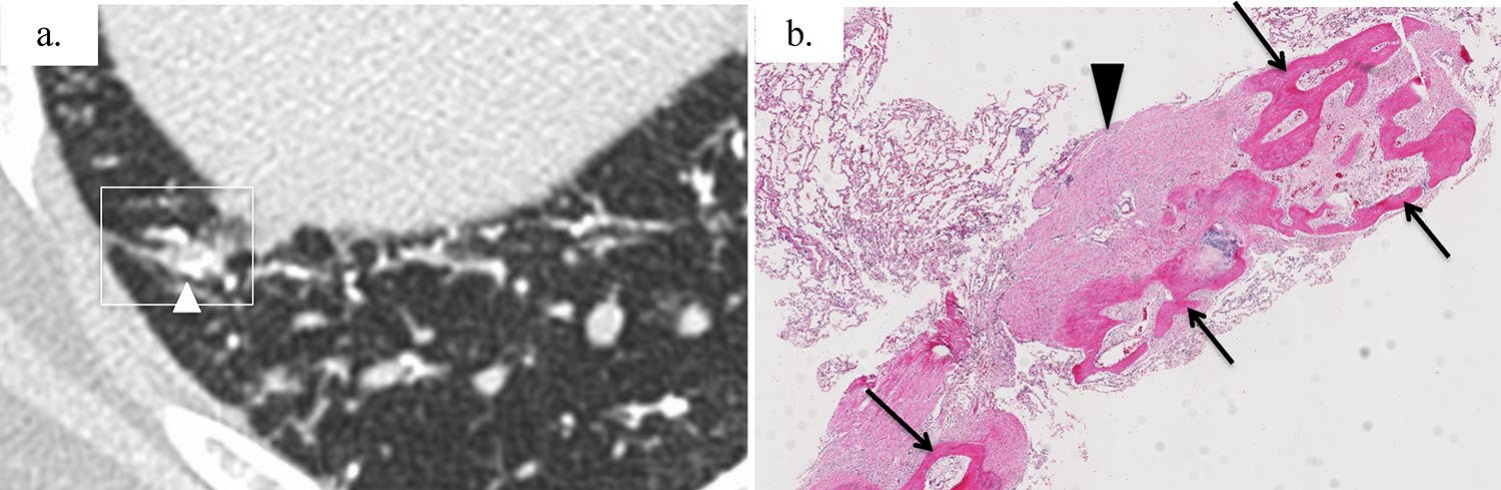
Precise CT–pathologic correlation of a 29-year-old man of idiopathic DPO; the side-by-side comparison of local CT findings at the surgical lung biopsy resection site indicated by a white square (**a**) and histological images. **a** Unenhanced axial chest CT in lung window settings showing band-like opacity (arrowhead) in the subpleural area. **b** Specimen showing ossification (arrows) containing fatty bone marrow. Relatively large-sized cicatricial OP (arrowhead) and partial ossification are also observed. Focus of cicatricial OP (arrowhead) and ossification (arrows) are mixed with each other (× 20, HE stain)

The CT findings of band-like opacity correlated with the lung specimens obtained from the three cases. The biopsy specimens revealed a relatively large cicatricial OP and partial ossification (Fig. 7). The CT findings of GGA correlated with those of the lung specimens obtained from one case. Areas with GGA histologically corresponded to hemorrhage or alveolar septal thickening.

### Prevalence in a cohort of screening metastasis from extrathoracic malignancies

27901CT examinations were performed on 8111 cases (4411 men, 3700 women; mean age, 73 years; range: 18–90 years). Of these, 3921 cases were smokers. 283 cases (3.5%) had ILAs; 43 were non-subpleural, 140 were subpleural non-fibrotic, and 100 were subpleural fibrotic. Of these, DPO was seen in a total of 26 cases (0.3% of all cases, 9.2% of ILAs cases), with 1 case of non-subpleural, 17 cases of subpleural non-fibrotic, and 8 cases of subpleural fibrotic. All the 26 cases with DPO showed a mixture of branching, linear, and round structures with or without high attenuation.

## Discussion

This is the first report of precise CT–pathological correlation of idiopathic DPO and its prevalence in population-based cohort. In idiopathic DPO, branching, round, and linear structures with or without high attenuation are major HRCT findings, and not all lesions showed bone-like high attenuation at bone window setting CT images. These CT lesions histologically corresponded not only to dendriform ossification but also to cicatricial OP or fibrosis. Furthermore, in our population-based cohort, we found DPO in 0.3% (26 of 8111 cases) of all cases and 9.2% (26 of 283 cases) of ILAs cases, respectively.

In two of the 16 cases of idiopathic DPO in the present study, despite the histologic evidence of bone, no high attenuation structure was identified on CT corresponding to the biopsy site. The reasons why CT of idiopathic DPO shows iso- and low-attenuation levels in the bone window setting could be due to the small size of ossification component, fatty marrow inside the ossification, and a mixture of other components such as cicatricial OP, fibrosis, and ossification. The ossification size in the biopsy specimen in this study was a minimum of 0.1 mm, and very small ossification may not be identified on HRCT due to the limitations of the spatial resolution. The pixel size, which is approximately 0.65 mm at a field of view of 30 cm, and the 1- or 2-mm slice thickness will result in a decrease in the attenuation value of bones smaller than 0.6 mm by a partial volume effect. In a previous study, Kim et al. reported that bone could be correctly identified in the special condition of the osteoporosis window settings in cases with DPO with a background of UIP and NSIP [12]. Under general bone window settings (WW 2500HU, WL 500HU), small ossification or ossification with a large proportion occupied by bone marrow may not have sufficiently high attenuation values to be identified as calcification owing to partial volume effects. In other words, areas with no high-attenuation structures on CT sometimes include ossification, even if it does not seem to contain ossification. It is essential for the diagnosis of Idiopathic DPO that linear or branching structures with no high attenuation on CT is sometimes seen.

The mechanisms of growth of pulmonary ossification have not been fully understood. Ectopic ossification is hypothesized to occur as a result of various factors, such as preceding lung injury and an alkaline environment. Lung tissue injury causes an alkaline environment, and calcium salt precipitation in hypoxic and alkaline environments promotes the activation of alkaline phosphatase, which in turn activates fibrogenic cytokines. In addition, transforming growth factor-β, which plays a role in promoting tissue regeneration and fibrosis in response to epithelial injury, is produced and stimulates chondroblasts and osteoblasts, which are also believed to lead to ossification [16].

Recently, DPO has been described as a part of histological findings of cicatricial OP and was thought to be a sequela of OP [5]. The imaging findings of idiopathic DPO may reflect different phases of cicatricial OP: ossification as the end of cicatricial OP, cicatricial OP as the precursor of ossified nodules, and fibrotic lesions associated with surrounding lung injury. In our study, histological evaluation identified ossification at the periphery of the cicatricial OP in most cases, and ossification was widely attached to cicatricial OP. There was also a gradual change from cicatricial OP to ossification in the nature of the ossification, suggesting that ossification arose against the background of cicatricial OP. It is possible that bone may develop in the future from cicatricial OP lesions that do not show ossification at the time of evaluation. In this study, the branching, linear, and round structures of CT of DPO cases corresponded to ossification, cicatricial OP, and fibrosis mainly in the alveolar ducts and air spaces. Im et al. clarify the histological background of CT findings of pulmonary tuberculosis, and showed that a tree-in-bud appearance on CT corresponded to caseation necrosis within the alveolar duct and the respiratory bronchioles [17]. This tree-in-bud pattern of mycobacterial infections resembles the branching structure of DPO. Though the nature of the lesions is different: one is cicatricial OP and fibrosis or bone, the other is necrosis and granulomatous changes associated with tuberculosis, since they were similarly located in the respiratory bronchioles and alveolar duct, they might exhibit similar branching structures.

DPO has been reported to occur in elderly men and appears to be associated with chronic aspiration of gastric acid by Gruden et al. [18]. The distribution of CT findings in our cohort was similar to their reports, with lower and posterior predominance. Although their cohort included relatively older cases and did not match the age distribution of our cohort, it is possible that chronic aspiration of gastric acid may influence the formation of cicatricial OP and idiopathic DPO.

In this population-based cohort, we found DPO in 0.3% (26 of 8111 cases) of all cases and 9.2% (26 of 283 cases) of ILAs cases, respectively. DPO is thought to be one of the pathologic phenotypes in ILAs cases. In this cohort, DPO on CT was defined as lower lobe predominant linear, branching, or dot-like structures with calcification at least in one lesion. However, the present study demonstrates the possibility that DPO represent iso-attenuated linear, branching, or dot-like structures without calcification on CT. These diagnostic criteria cannot catch up such cases. Therefore, idiopathic DPO may be more frequent.

UIP sometimes has ossification in the pathological findings [12, 19] and is the most frequent pathological form in ILAs [1]. Thus, it is possible that bone found in ILAs might reflect the bone associated with UIP. However, from the present and previous studies [6–10], CT findings of idiopathic DPO are quite different from those of ossification in UIP. The former is linear, branching or round structures sometimes with calcification while the latter is calcification in reticulation.

Our study has some limitations. First, because of the retrospective and multi-institutional nature of the study, scanning protocols were not standardized. Although cases with a slice thickness of ≤ 2 mm were included in this study, it may be necessary to consider using CT with even higher resolution, such as ultra-high-resolution CT, in the future. Second, the evaluation of attenuation levels on CT at the bone window settings was subjective and not quantitative. Third, some CT findings, such as pleural indentation, could not be evaluated with side-by-side pathological correlation because biopsy specimens did not have appropriate sites. Fourth, we could not figure out the reason that only the branching s**t**ructure**s** showed high attenuation. Finally, due to using strict diagnostic criteria, the prevalence of DPO may be larger than that in the present cohort. Namely, the cases with relatively low attenuated ossification on CT might not be counted as the cases with ossification.

In conclusion, idiopathic DPO is one of the pathologic phenotypes of ILAs. In idiopathic DPO, branching, round, and linear structures with or without high attenuation are major HRCT findings, and not all lesions showed bone-like high attenuation in bone window CT images. These CT lesions histologically corresponded to not only dendriform ossification but also cicatricial OP or fibrosis. Ossified lesions sometimes did not show high attenuation on CT of bone conditions, suggesting the effects of its small size and the partial volume effect of bone marrow fatty tissue.

### Supplementary Information

Below is the link to the electronic supplementary material.

Supplementary file1 (DOCX 43 KB)
