# Supplementary material for: Idiopathic dendriform pulmonary ossification as the phenotype of interstitial lung abnormalities: CT–pathologic correlation and prevalence
Source: Jpn J Radiol. 2024 May 14;42(9):993–1002. doi: 10.1007/s11604-024-01590-8 (PMC11364601; doi:10.1007/s11604-024-01590-8)
Supplement: Supplementary file 1 — Supplementary file1 (DOCX 43 KB) [file 11604_2024_1590_MOESM1_ESM.docx]

**Electronic Supplemental Materials**

**Detailed definitions of each term**

Branching was defined as a dendritic structure, linear as a linear or unbranched cylindrical structure, and round as small nodules or individual short rod-like opacities. Band-like opacities were defined as the structures seen as thick linear opacities extending in a radial manner along the line of a bronchus toward the pleura or occurring in peripheral locations bearing no relationship to the bronchi [11]. Lobules with decreased attenuation were defined as focal zones of decreased attenuation corresponding to secondary pulmonary lobules [12]. Pleural indentation was defined as a linear area in contact with the pleura. Other CT findings were defined according to the Fleischner Society glossary of terms for thoracic imaging [13]. These CT findings were selected for analysis according to a preliminary review of the CT images and previous case reports [4-8]. Subcategories of ILAs were defined as follows: non-subpleural as ILAs without predominant subpleural localisation, subpleural non-fibrotic as ILAs with a predominant subpleural localisation and without evidence of fibrosis, subpleural fibrotic as ILAs with a predominant subpleural localisation and with evidence of pulmonary fibrosis [1].

Supplemental table 1. Summary of κ value and agreement

| CT findings | κ value | Agreement (%) |
| --- | --- | --- |
| High | 1 | 100 |
| Branching | 1 | 100 |
| Linear | N.A. | 100 |
| Round | N.A. | 100 |
| Iso | 1 | 100 |
| Branching | 1 | 100 |
| Linear | 0.84 | 93 |
| Round | 1 | 100 |
| Low | 0.75 | 87 |
| Branching | 0.9 | 93 |
| Linear | 0.63 | 93 |
| Round | 1 | 100 |
| Pleural indentation | 0.73 | 87 |
| Lobules with decreased attenuation | 0.73 | 87 |
| Band-like opacity | 1 | 100 |
| Ground-glass attenuation | 0 | 93 |
| Reticulation | N.A. | 100 |
| Cyst | N.A. | 100 |
| Craniocaudal distribution |  |  |
| Upper predominance | N.A. | 100 |
| Lower predominance | 0.85 | 93 |
| Transaxial distribution |  |  |
| Central predominance | N.A. | 100 |
| Peripheral predominance | 0.87 | 93 |
| Anteroposterior distribution |  |  |
| Anterior predominance | N.A. | 100 |
| Posterior predominance | 0.85 | 93 |

N.A.=not applicable
